# Screening and Selection of a New Medium and Culture Conditions for Diosgenin Production via Microbial Biocatalysis of SYt1

**DOI:** 10.3390/bioengineering11111098

**Published:** 2024-10-31

**Authors:** Shiyao Han, Yiyu Zhao, Fangyuan Mou, Zhen Yang, Ningxiao Li, Mengqi Cheng, Heshaungyi Xie, Baofu Qin, Young Tang

**Affiliations:** 1 Shaanxi Centre of Stem Cells Engineering & Technology, Key Laboratory of Livestock Biology, College of Veterinary Medicine, Northwest A&F University, Yangling 712100, Chinazhaoyiyu@nwafu.edu.cn (Y.Z.); youngzhen@nwafu.edu.cn (Z.Y.);; 2 College of Life Science, Northwest A&F University, Yangling 712100, China

**Keywords:** diosgenin, liquid fermentation products, endophytic *Bacillus licheniformis*, microbial biocatalysis, media optimization

## Abstract

Diosgenin (DSG) is a phytosterol saponin mainly found in *Dioscorea zingiberensis* C.H. Wright. It has shown promising results in treating various diseases such as cancer, diabetes, arthritis, asthma, and cardiovascular diseases. Diosgenin is also an important medicinal chemical for synthesizing various steroid medicines. The production of diosgenin by acid hydrolysis generates a large amount of wastewater, leading to severe environmental pollution. However, producing diosgenin through microbial fermentation can effectively reduce environmental pollution. Numerous studies have demonstrated that various microorganisms can produce diosgenin via solid-state fermentation. Nevertheless, due to the complexity, high maintenance costs, uneven heat production, and other characteristics of solid-state fermentation, it is not commonly used in the industrial production of diosgenin. In contrast, liquid fermentation offers advantages such as simple operation, easy maintenance, and stable fermentation, making it more suitable for the industrial production of diosgenin. However, few studies have focused on producing diosgenin using liquid fermentation. In this study, endophytic *Bacillus licheniformis* SYt1 was used to produce diosgenin via liquid fermentation, with *Dioscorea* tuber powder as a substrate. Soxhlet extraction and silica gel column chromatography were employed to identify the diosgenin from the liquid fermentation products. Suitable fermentation conditions were screened and identified. The environmental variables that significantly affect the diosgenin yield were determined by the Plackett–Burman design (P-BD) with eight factors. The three factors (peptone, yeast extract powder and inorganic salt) with the greatest influence on the diosgenin yield were selected and further optimized using a response surface methodology (RSM). The final culture conditions were determined to be 35.79 g/L of peptone, 14.56 g/L of yeast extract powder, and 1.44 g/L of inorganic salt. The yield of diosgenin under these conditions was 132.57 mg/L, which was 1.8 times greater than the yield under pre-optimization conditions. This effective, clean, and promising liquid fermentation method possesses the potential to replace the traditional acid hydrolysis method for the industrial production of diosgenin.

## 1. Introduction

Diosgenin is a natural steroidal compound widely present in medicinal plants, including *Dioscorea zingiberensis*, *Trigonella foenum-graecum*, and *Dioscorea panthaica* [1]. The compound’s molecular formula is C_27_H_42_O_3_, it has a molecular mass of 414.621, and it melts between 204 and 207 °C. Diosgenin is characterized by a crystalline structure and manifests as white needle-like crystals or light powder. It exhibits minimal solubility in water but is readily soluble in organic solvents such as petroleum ether and chloroform. It is the primary raw material for steroidal hormone drugs, including progesterone, androgens, and contraceptives [2,3,4,5]. Furthermore, diosgenin possesses medicinal value, demonstrating efficacy in the treatment of leukemia, hypercholesterolemia, rheumatoid arthritis, and cancer. It is also an active ingredient in numerous clinical drugs, including anti-tumor, anti-inflammatory, blood sugar regulation, and immunomodulatory medications [6,7,8,9,10]. Consequently, with the advancement of the pharmaceutical industry, a considerable quantity of diosgenin is employed in drug production.

Currently, over 400 drugs are produced using diosgenin as a raw material. International market demand is 6000 tons per year, increasing annually by 8–10%. *Dioscorea zingiberensis* is the primary raw material for diosgenin production in China. It has an annual output of approximately 3000 tons, accounting for 80% of the world’s total. Due to supply shortages, diosgenin prices are as high as 650,000 to 700,000 yuan [3]. The low content of diosgenin analogs in plant tissues and the high costs of the subsequent structural modification and chemical synthesis make it crucial to find methods to effectively increase diosgenin yield [11].

Diosgenin is traditionally produced by direct acid hydrolysis. A large amount of strong acid is used in this process to dissolve the cell walls and saponin bonds, allowing diosgenin to be released. This method has a low yield and causes severe environmental pollution. Researchers found that using microbes from plants to hydrolyze starch or cellulose before performing acid hydrolysis on saponins significantly improved the yield of diosgenin. Enzymatic hydrolysis involves adding specific amylases or cellulases for preliminary hydrolysis, followed by strong acid to break the saponin bonds. Research shows that using cellulase and α-amylase to enzymatically hydrolyze *Dioscorea zingiberensis* significantly increases the subsequent diosgenin yield [12]. While these methods increase yields, they still use strong acids, which cause environmental pollution. Microbial transformation is a promising method. Microbes produce various enzymes during fermentation that release diosgenin and further cleave glycosidic bonds in saponins to form diosgenin. Numerous studies show that microbes can convert saponins into diosgenin. *Gibberella intermedia* WX12 can convert saponins in *Dioscorea zingiberensis* to diosgenin. Co-fermentation with *Gibberella intermedia*, *Fusarium* (CPCC 400709), and *Septoria* (CPCC 400737) yields 2.79% diosgenin [13]. *Trichoderma reesei* can produce diosgenin by degrading the rhamnose or glucose attached to the C-3 position of saponins [14].

Most studies on microbial transformation for diosgenin production use solid-state fermentation. However, solid-state fermentation is complex to operate, costly to maintain, and has an uneven heat distribution, making it unsuitable for the industrial production of diosgenin. In contrast, liquid fermentation is simpler to operate and maintain, and offers stable fermentation, making it more suitable for industrial production. Currently, there is little research on using liquid fermentation to produce diosgenin. Additionally, the fermentation medium and conditions are crucial factors affecting the fermentation process, impacting microbial metabolism and the final product yield. Therefore, exploring the optimal conditions for microbial metabolism during fermentation is essential to increase the final product yield.

The SYt1 strain, isolated from *Dioscorea*, has been found in preliminary experiments by our lab to catalyze the conversion of diosgenin saponins to diosgenin. This strain can be used in biotransformation processes to produce diosgenin. However, the effects of medium composition and fermentation conditions on SYt1’s ability to produce diosgenin during fermentation are not well understood. Therefore, this study aims to identify the optimal medium components and fermentation conditions for SYt1 through single-factor and multi-factor experiments to enhance the final yield of diosgenin.

This study presents an environmentally friendly and straightforward method for producing diosgenin using liquid fermentation with SYt1. It also identifies the optimal fermentation conditions for SYt1. The findings provide preliminary data support for industrial-scale production and have the potential for application in the commercial production of diosgenin.

## 2. Materials and Methods

### 2.1. Microorganisms

Endophytic *Bacillus licheniformis* SYt1, isolated from *Dioscorea zingiberensis* C. H. Wright (DZW), was utilized in liquid fermentation. This strain, with patented potential, convert DZW into diosgenin. It is deposited at the China General Microbiological Culture Collection Center (https://www.cgmcc.net/ accessed on 5 October 2024).

### 2.2. Preparation of Inoculum

The strain was plated on recovery medium (3 g/L beef extract, 10 g/L peptone, and 5 g/L NaCl). The flask was placed in a thermostatic rotary shaker (CHA-S-Guohua, JiangSu, China) at 28 °C, 180 rpm for 24 h. After diluting the activated bacterial solution, 100 μL was transferred to the soil inoculum culture medium and incubated at 28 °C for 48 h. Well-grown single colonies were selected for subsequent fermentation experiments.

### 2.3. Biotransformation of DZW to Diosgenin by Liquid Fermentation

Dried *Dioscorea* roots were ground into powder by a grinder (FW100, Changzhou Jintan Youlian Instrument Research Institute, Changzhou, China). The powder was sifted through a 100-mesh sieve and stored at 4 °C. An amount of 100 mL liquid fermentation medium (containing 10 g/L *Dioscorea* rhizome powder, 15 g/L glucose, 10 g/L peptone, 5 g/L NaCl, 1 g/L K_2_HPO_4_) was added to a 250 mL flask. A single colony from the inoculated medium was transferred to the liquid fermentation medium, 28 ° C, 180 r/min incubation for 6 days. Aliquots of 150 mL of fermentation broth were poured into 500 mL round-bottom flasks in batches, 250 mL of petroleum ether added, and Soxhlet extraction performed at 85 °C. The concentration was refluxed for 4 h, and the extraction was repeated three times to ensure thorough extraction. The upper extract was concentrated at 85 °C and dried to yield crude diosgenin.

### 2.4. Purification of Liquid Fermentation Production

Chromatographic silica gel was loaded into the column using the wet loading method. The obtained sample was mixed with the silica gel mixture and subsequently loaded into the column along with the eluent, which in this case was cyclohexane. The pressure in the column had to be increased to maintain a steady flow rate of 18 mL per minute. The conical flask was changed for every 100 mL of eluent collected to gather purified products. The Thin-Layer Chromatography (TLC) method was used to identify the eluent containing diosgenin. The diosgenin-containing fractions were combined with rotary evaporators and dried.

### 2.5. Determination of Diosgenin by Thin-Layer Chromatography

The samples collected in Section 2.5 were applied to a TLC plate using a 0.3 mm glass capillary spotter. The TLC plate was placed vertically in the developing solvent tank containing cyclohexane and ethyl acetate (*v*:*v* = 4:1). When the liquid level was 1 cm from the top edge, it was removed and dried. Subsequently, it was immersed in 10% sulfuric acid ethanol developer for 2 s and dried.

### 2.6. Determination of Diosgenin by HPLC

An amount of 0.1 g of the dried sample was added to 10 mL of methanol and heated by ultrasound for 15 min to accelerate dissolution. After complete dissolution, samples were filtered through a 0.45 micron membrane and transferred to vials for liquid chromatography analysis. The high-performance liquid chromatography (HPLC) analysis was run on an Agilent 1290 HPLC system with an auto-sampler (Agilent Technologies, Santa Clara, CA, USA). The HPLC system was equipped with an HPLC column (XTERRA MS C18, 4.6 × 250 mm, 5 μm; Waters; MA, USA). The column temperature was 25 °C. The wave length of the UV detector was 210 nm. The loading quantity of the samples was 10 µL. Acetonitrile/water = 8:2 was the eluent (1.0 mL/min). The yield of diosgenin was calculated with the following Equation (1):

Diosgenin yield (%) = diosgenin production (mg/mL)/DZW (mg/mL) × 100%(1)

### 2.7. Determination of Diosgenin by Fourier-Transform Infrared

Comparisons of the Fourier-transform infrared (FT-IR) spectrum of the obtained diosgenin-like crystal product with the diosgenin standard product were screened by a Bruker vertex70 (AVANCEIII500, Switzerland, Germany) infrared spectrometer system. The crystal product and standard product were both dried for 2 days prior to FT-IR measurement. Round transparent tablets containing the crystal product sample or diosgenin standard product were subjected to 32 scans with a 4 cm^−1^ resolution in the region of 600–4000 cm^−1^ by the Bruker vertex 70 infrared spectrometer. An attenuated total reflectance (ATR) sampling accessory with a diamond window was used for measurements.

### 2.8. Nuclear Magnetic Resonance Method

The diosgenin standard and sample (10 mg) were added to nuclear magnetic resonance tubes with a diameter of 5 mm and dissolved in 0.6 mL of chloroform-d using sonication. 1H-NMR, 13C-NMR, and distortionless enhancement by polarization transfer (DEPT) were carried out on a nuclear magnetic resonance spectrometer with tetramethylsilane (TMS) as the standard material.

### 2.9. Effect of Carbon Source on Diosgenin Production

The liquid fermentation medium was modified by replacing glucose with different carbon sources (soluble starch, lactose, dextrin, sucrose, inulin, or fructose) at a concentration of 20 g/L. After fermentation, the diosgenin content was detected using the HPLC.

### 2.10. Effect of Nitrogen Source on Diosgenin Productio

The nitrogen source in the medium was screened using 10 g/L yeast extract, corn flour, soybean meal, peptone, tryptone, ammonium chloride, and ammonium acetate in the replacement of the original nitrogen source. The subsequent method is the same as Section 2.9.

### 2.11. Effect of Surfactant on Diosgenin Production

In order to detect the effect of the surfactant on diosgenin production, Tween-80, glycerol, choline and polyethylene glycol were added to liquid fermentation medium as the experimental group. The subsequent method is the same as Section 2.9.

### 2.12. Selection of Significant Factors by the Plackett–Burman Design

The Plackett–Burman design (P-BD) was used to screen the variables that significantly affected the transformation of diosgenin by the fungus. A single-factor test was performed to determine the low and high levels of each variable, and then P-BD (Design Expert v8.0.6) was used to evaluate the seven factors. The contents included (Table 1) yeast extraction (g/L), peptone (g/L), sucrose (g/L), dextrin (g/L), *Dioscorea* rhizome powder (g/L), inorganic salt (g/L), fermentation period (day), inoculum age (h), and virtual item1/2/3, which are represented as A, B, D, E, G, H, K, L, C, F, and J. The diosgenin yield (%) was determined by calculating the average of three replicates of independent measurements. The significant variables obtained by this method were used for the further optimization of biological processes.

### 2.13. Bioprocess Optimization by Box–Behnken Design

A Box–Behnken design (BBD) analysis was carried out to determine the significant influence on the biotransformation of DZW and the interaction between the selected factors. The optimal value for each variable that has a significant influence on the formation of diosgenin was further identified in order to maximize the yield of diosgenin. As shown in Table 2, three factors selected from the PBD for further optimization were peptone (g/L), yeast infusion powder (g/L), and inorganic salt (g/L), which were denoted A, B, and C, respectively.

### 2.14. Verification Experiments

Based on the optimal values obtained from the PBD-BBD, the medium and culture conditions were optimized. The accuracy of the model can be ensured by using high-performance liquid chromatography (HPLC) to determine the conversion rate of diosgenin after optimization.

### 2.15. Statistical Analysis

All experiments were performed in three replicates, and the data consisted of the means of independent measurements. The results were presented as the mean ± S.D. for three replicates. Design-Expert software V8.0.6.1 (Minneapolis, MN, USA) was utilized for the statistical analysis and graph plotting. *p* < 0.05 was considered to be significant.

## 3. Results

### 3.1. Identifcation of Diosgenin Product in Fermentation Broth

**Figure 1:**
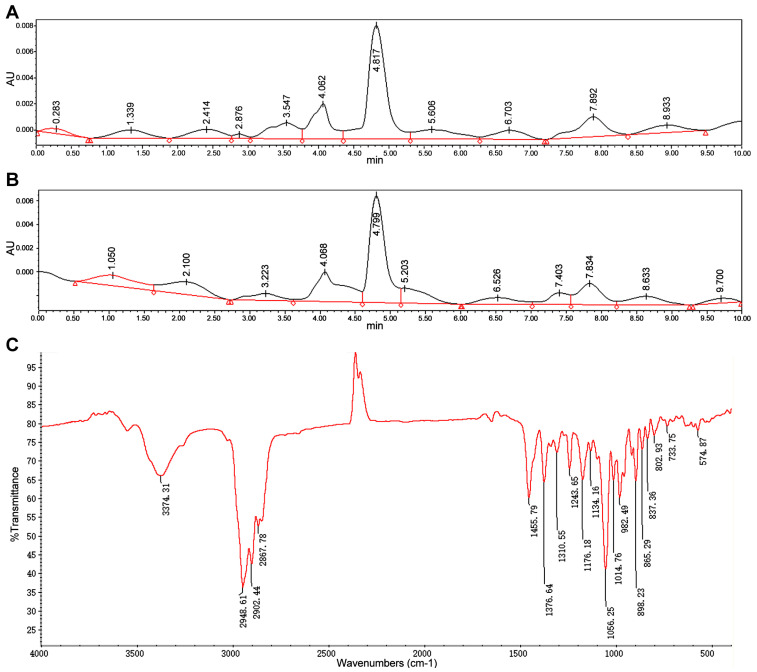
Analysis of biotransformation products converted by SYt1. (**A**) Chromatograms of diosgenin standard by HPLC. (**B**) Chromatograms of fermentation sample by HPLC. (**C**) FTIR spectrum of the fermented sample.

**Figure 2:**
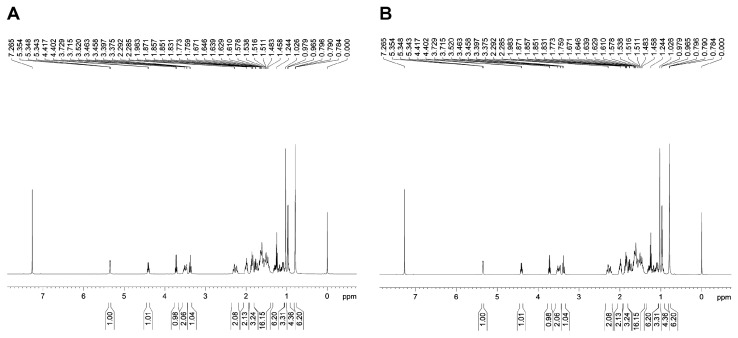
Detection of diosgenin by nuclear magnetic resonance. (**A**) NMR carbon spectrum of fermentation sample. (**B**) NMR hydrogen spectrum of fermentation sample.

This study utilized thin-layer chromatography (TLC), high-performance liquid chromatography (HPLC), infrared spectroscopy (IR), and nuclear magnetic resonance (NMR) to confirm whether *Bacillus licheniformis* SYt1 can produce diosgenin from *Dioscorea* rhizome through liquid fermentation. The TLC results showed that column-purified samples had a distinct purple spot under UV light, the same as the diosgenin standard. The dried and purified product from the fermentation liquid had uniform white needle-like crystals without impurities. HPLC and IR spectroscopy were used to verify the substances produced by SYt1 in liquid fermentation. According to Figure 1, the sample peaks were formed at the same time as the diosgenin standard, which was 14 min. The NMR results indicated that the purified product contained 27 carbon atoms and 42 hydrogen atoms, consistent with the standard molecular formula of diosgenin (Figure 2). These findings demonstrated that SYt1 can utilize *Dioscorea* rhizome powder in liquid fermentation to produce diosgenin

### 3.2. Selection of Optimal Carbon Source for Diosgenin Production

**Figure 3:**
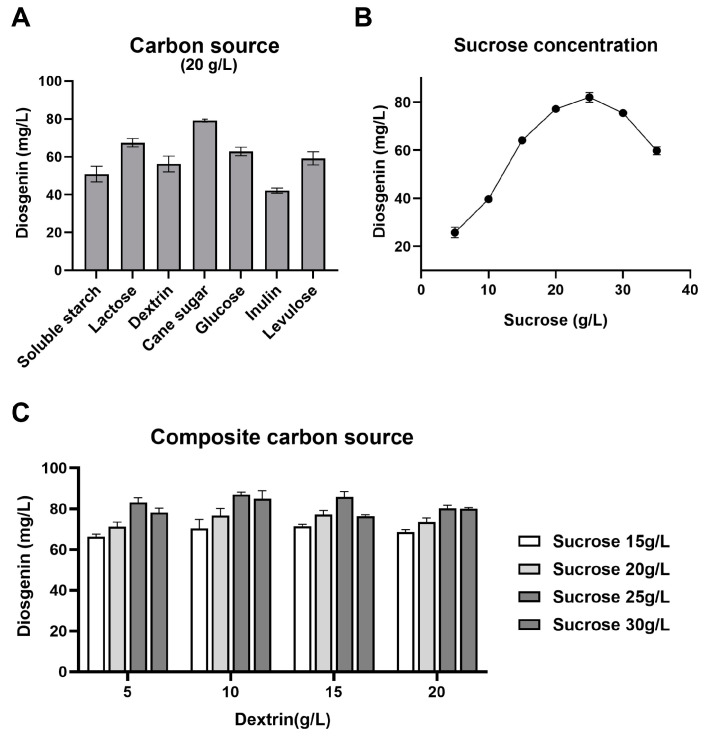
The influence of the carbon source on the participation of SYt1 in the biotransformation of DZW to diosgenin. (**A**) The influence of different carbon sources on the yield of diosgenin. (**B**) The influence of sucrose concentration on the yield of diosgenin. (**C**) The influence of different composites of carbon sources on the yield of diosgenin. *n* = 6–8 each group. Values are mean ± SD.

The first aim of this study was to evaluate the effects of different carbon sources on the production of diosgenin. As shown in Figure 3A, the different type of carbon source significantly affects the yield of diosgenin. Compared to polysaccharide-containing media, disaccharide-containing media generally promoted diosgenin yields. Using sucrose as the carbon source for SYt1 improved the yield of diosgenin production (79.07 mg/L), which was significantly higher than in the other six groups, in the fermentation process. Among polysaccharides, dextrin was the most suitable carbon source for SYt1. Further gradient tests indicated that different sucrose concentrations influenced the final diosgenin yield (Figure 3B). The highest yield of diosgenin (82.03 mg/L) was achieved at a sucrose concentration of 25 g/L. Rapid carbon sources promote early bacterial growth, while slow-acting sources sustain metabolic activity. Therefore, the effect of mixed carbon sources on the diosgenin yield was explored by adding both sucrose and dextrin. As shown in Figure 3C, mixed carbon sources affected the final diosgenin yield. The highest yield (86.93 mg/L) was achieved with 25 g/L sucrose and 10 g/L dextrin, surpassing single carbon sources.

### 3.3. Selection of Optimal Nitrogen Source for Diosgenin Production

**Figure 4:**
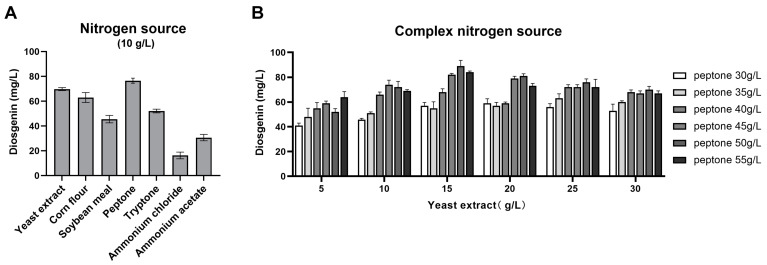
The influence of the nitrogen source on the participation of SYt1 in the biotransformation of DZW to diosgenin. (**A**) The influence of different nitrogen sources on the yield of diosgenin. (**B**) The influence of different composites of nitrogen sources on the yield of diosgenin. *n* = 6–8 each group. Values are mean ± SD.

During fermentation, the nitrogen source directly affects the growth and development of the strain, thereby influencing the yield of metabolic products. This study investigated the impact of different nitrogen sources on the diosgenin yield. The results indicated that using organic nitrogen sources increased diosgenin production compared with using inorganic nitrogen sources. There was a greater increase in diosgenin production from nitrogen sources with the largest protein molecules (soybean meal, peptone, tryptone) than from nitrogen sources with smaller protein molecules (yeast extract, corn steep liquor). Peptone was the most effective nitrogen source for increasing the yield of diosgenin, followed by yeast extract (Figure 4A). The effect of mixed nitrogen sources on diosgenin production was further explored. As shown in Figure 4B, fermentation with mixed nitrogen sources of yeast extract and peptone significantly enhanced the diosgenin yield compared to single nitrogen sources. The highest diosgenin yield (89.00 mg/L) was achieved with 50 g/L peptone and 15 g/L yeast extract.

### 3.4. Selection of Optimal Liquid Fermentation Medium Components for Diosgenin Production

**Figure 5:**
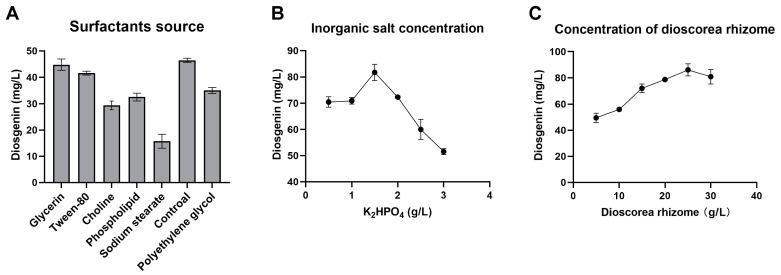
The influence of the media composition on the participation of SYt1 in the biotransformation of DZW to diosgenin. (**A**) The influence of different nitrogen sources on the yield of diosgenin. (**B**) The influence of different composites of inorganic salt on the yield of diosgenin. *n* = 6–8 each group. Values are mean ± SD. (**C**) The influence of different concentrations of dioscorea rhizomeon the yield of diosgenin. *n* = 6–8 each group. Values are mean ± SD.

Single-factor experiments were conducted to detect the important factors that affect the SYt1-catalyzed conversion of *Dioscorea* powder to *Dioscorea* saponins. The composition of the medium is crucial for microbial activities. It may affect the conversion of *Dioscorea* rhizome powder to diosgenin by the biotransformation of SYt1 strains. Thus, this study further explored the impact of surfactant types, inorganic salt concentrations, and substrate concentrations on the diosgenin yield. In comparison to the control group, adding surfactants such as Tween-80, sodium stearate, choline, and polyethylene glycol 6000 significantly reduced the diosgenin yield; however, adding glycerol had no significant effect (Figure 5A). As shown in Figure 5C, when the concentration of *Dioscorea* rhizome powder was below 25 g/L, the diosgenin yield increased with the increase in powder concentration. However, when the concentration exceeded 25 g/L, further increases in powder concentration reduced the final diosgenin yield. Inorganic salt concentrations showed a similar trend. The optimal concentration of inorganic salt was 1.5 g/L, resulting in a diosgenin yield of 81.71 mg/L.

### 3.5. Selection of Optimal Liquid Fermentation Conditions for Diosgenin Production

**Figure 6:**
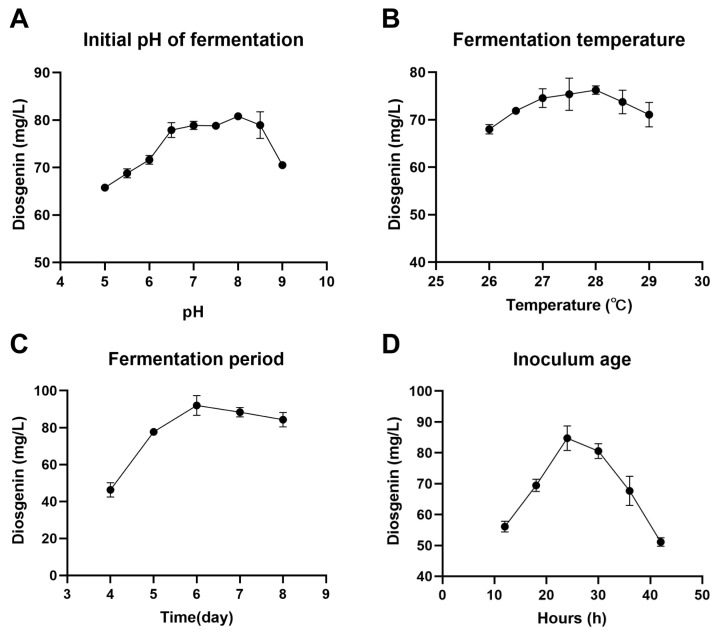
Influence of different pH on yield of diosgenin. (**A**) Influence of different temperature on yield of diosgenin. (**B**) Influence of different fermentation period on yield of diosgenin. (**C**) Influence of different inoculum age on yield of diosgenin. (**D**) Influence of different inoculum age on yield of diosgenin.

A suitable fermentation environment can enhance microorganisms’ activity. To determine the optimal conditions for SYt1 to convert *Dioscorea* rhizome powder to diosgenin in fermentation, this study explored the effects of initial pH, fermentation temperature, fermentation duration, and inoculum age on the diosgenin yield. The results showed that a slightly alkaline environment (pH 7.5–8.5) was conducive to metabolic activity and increased the diosgenin yield to 80.79 mg/L (Figure 6A). As shown in Figure 6B, temperature changes also significantly affected the conversion of the *Dioscorea* rhizome to diosgenin by SYt1. The optimal temperature for diosgenin production was 28 °C. It was observed that temperatures above or below this range resulted in a reduction in the final yield. The activation time of the inoculum is another important consideration. It was observed that a 24 h activation period resulted in significantly higher yields of diosgenin compared to other activation times. The results of fermentation duration experiments indicated that the highest diosgenin content was reached on the sixth day, with no further increase upon extending the fermentation time (Figure 6C).

### 3.6. Screening the Main Effect Factors on Diosgenin Production by Plackett–Burman Design

The Plackett–Burman (P-B) design was employed to investigate the influence of various factors (yeast extract, peptone, sucrose, dextrin, *Dioscorea* rhizome powder, inorganic salt concentration, fermentation time, and inoculum age) on the diosgenin yield in the fermentation process. The experimental results, shown in Table 3, indicated that the various factors significantly impact the diosgenin yield. The highest response was observed in run 1, followed by runs 9, 10, and 11. The addition of elevated concentrations of yeast extract and sucrose to the medium resulted in a notable increase in diosgenin production. Conversely, the lowest response was observed in run 5, whereby elevated inorganic salt concentrations within the medium resulted in a reduction in diosgenin yields. Overall, these results suggested that increasing the concentrations of carbon and nitrogen sources is beneficial for enhancing metabolite production. However, the excessive addition of inorganic salts and *Dioscorea* rhizome powder can reduce metabolite yield.

**Figure 7:**
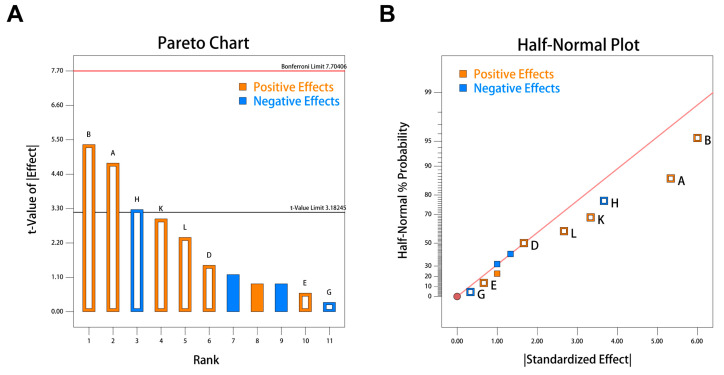
The PBD plots. (**A**) Pareto chart. (**B**) Half-normal probability plot. The positive effects and negative effects are, respectively, indicated by yellow points and blue points. Factors a, b, d, e, g, h, k, and l respectively represent yeast extract, peptone, sucrose, dextrin, *Dioscorea* rhizome powder, inorganic salt concentration, fermentation period, and inoculum age.

According to the analysis of variance (ANOVA) results from the Plackett–Burman design (PB) model (Table 3), yeast extract, peptone, and inorganic salt concentration are the main influencing factors, with *p*-values of 0.043. Additionally, the PBD model’s *p*-value of 0.043 < 0.05 indicates significant regression. The model’s R^2^ is 0.9403, meaning 94.04% of the experimental results can be explained by the model, and the adjusted R^2^ is 0.8328, indicating that the actual values closely match the model’s predictions. Therefore, these results suggest that the model is highly reliable and can be used to predict changes in diosgenin yield. Based on these findings, the final linear fitting model for diosgenin yield is given as Equation (2). The concentrations of peptone, yeast impregnating powder and inorganic salts exceeded t-value, indicating that the three were the main influencing factors(Figure 7A), All three of them had a positive impact(Figure 7B)

R1 = 98.17 + 2.67A + 3.00B + 0.83D + 0.33E − 0.17G − 1.83H + 1.67K + 1.33L(2)

### 3.7. Optimization of Fermentation Conditions for Diosgenin Production by Box–Behnken Design

The data were further analyzed by the BBD using Design-Expert software for a three-factor, three-level optimization experiment with peptone, yeast dipping powder, and inorganic salt concentration as experimental factors and diosgenin yield as the response value. The fitted model was obtained based on a variance analysis.

The results are also shown in Table 4. Peptone, yeast dipping powder, and inorganic salt concentration had a significant effect on the Y-value, with F-values of 10.95, 8.45, and 6.79 and *p*-values of 0.0130, 0.0227 and 0.0351 respectively. The interaction between peptone and yeast dipping powder had a significant effect on the yield of *Dioscorea* saponins with an F-value of 9.73004 and *p*-value of 0.0169. In addition, the BBD model had an F value of 34.32, *p* value <0.0001 and missing fit 0.2049 > 0.05. The R^2^, predicted R^2^ and adjusted R^2^ were 0.9778, 0.7587 and 0.9493 respectively, indicating that the model has high confidence and can be used to predict changes in *Dioscorea* saprophyllum yield.

**Figure 8:**
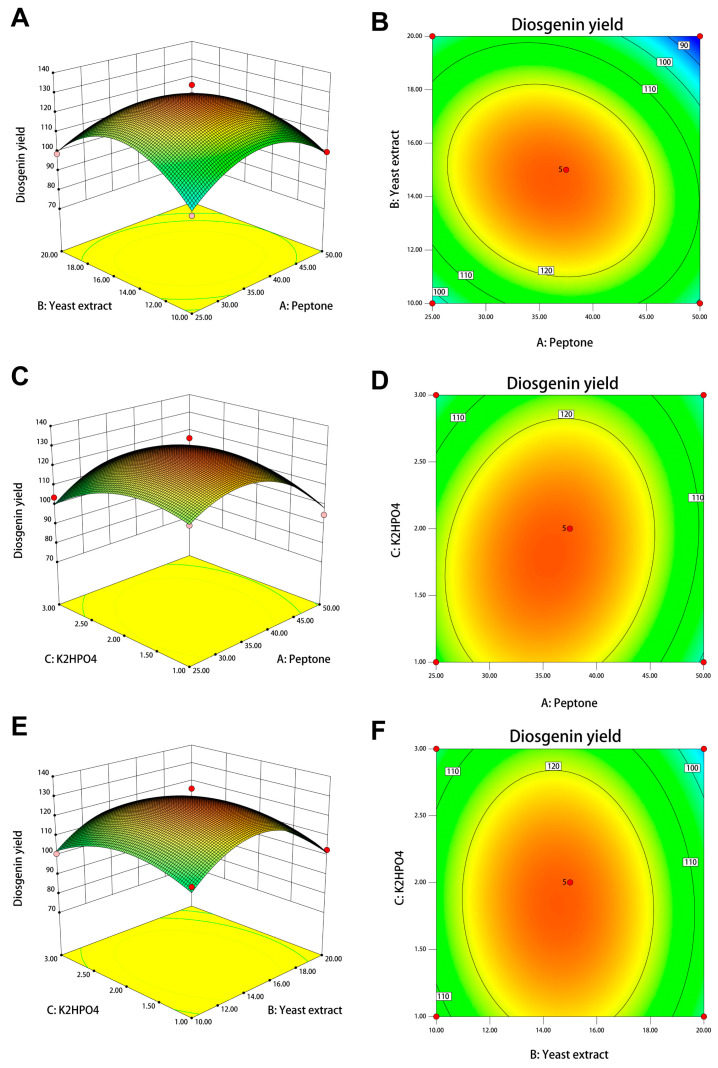
Mutual factor interactions among peptone, yeast extract, and K_2_HPO_4_ addition on diosgenin yield. (**A**) Three-dimensional response surface plot of mutual interaction between peptone and yeast extract. (**B**) Two-dimensional contour map of mutual interaction between peptone and yeast extract. (**C**) Three-dimensional response surface plot of interaction between peptone and K_2_HPO_4_ addition. (**D**) Two-dimensional contour map of mutual interaction between peptone and K_2_HPO_4_ addition. (**E**) Three-dimensional response surface plot of mutual interaction between yeast extract and K_2_HPO_4_ addition. (**F**) Two-dimensional contour map of mutual interaction between yeast extract and K_2_HPO_4_ addition.

As shown in Figure 8, the contour and response surface plots show the effect of the interaction of yeast dip with peptone (Figure 8A,B), inorganic salts with peptone (Figure 8C,D), and inorganic salts with yeast dip (Figure 8E,F) on the yield of *Dioscorea* saponins, respectively.

In conclusion, based on the results of the PBD-BBD analyses, the optimal medium composition was determined to be peptone 35.79 g/L, yeast dip 14.56 g/L, and inorganic salt concentration 1.81 times. The predicted yield of the diosgenin element for liquid fermentation using the optimum medium was 129.9 mg/L.

### 3.8. Experimental Validation

Verification experiments were conducted using the optimal culture conditions obtained from the PBD-BBD. The diosgenin content in the fermentation broth was determined by HPLC. The final diosgenin content was 132.57 mg/L, slightly higher than the predicted value of 129.9 mg/L. This represents an 80% increase in diosgenin yield compared to the pre-optimization fermentation conditions.

## 4. Discussion

This study confirmed the capability of SYt1 to produce diosgenin from *Dioscorea* rhizome powder using thin-layer chromatography, high-performance liquid chromatography, Fourier-transform infrared spectroscopy, and nuclear magnetic resonance. Subsequently, single-factor experiments were conducted to preliminarily determine the effects of changes in medium components and fermentation conditions on SYt1’s bioconversion of diosgenin. Finally, multifactor experiments and a response surface methodology were used to identify the optimal production conditions for SYt1’s bioconversion of diosgenin.

This study first optimized the culture conditions using single-factor analysis, focusing on nine aspects: carbon source, nitrogen source, substrate concentration, type of surfactant, inorganic salt concentration, seed age, fermentation time, fermentation temperature, and fermentation pH. In the screening process for the optimal carbon source, we found that sucrose was the most effective. This could be due to its relatively small molecular weight, allowing it to be quickly utilized by microorganisms [15]. Conversely, glucose was less effective as a carbon source, possibly due to the effects of catabolite repression [16]. Lactose, when hydrolyzed, produces galactose, which *Bacillus licheniformis* SYt1 cannot efficiently utilize, resulting in suboptimal performance as a carbon source [17,18]. Dextrin, inulin, and soluble starch [19] were also less effective, likely due to their larger molecular weights requiring hydrolysis before microbial absorption, thus hindering early microbial growth [19,20,21].

Carbon sources can be classified into fast-acting and slow-acting. Previous research has shown that using a composite carbon source consisting of both fast-acting and slow-acting carbon sources can enhance the enzyme production of *Bacillus licheniformis* HDYM-04 [22]. Similar results were found in this study, where a composite carbon source of sucrose and dextrin effectively increased the final yield of diosgenin. The composite carbon source provides suitable energy sources for both the early growth and later conversion stages of the strain, thereby improving the conversion efficiency. Therefore, in future fermentations, the continuous feeding method by Yangcun Sun can be adopted to extend the production of metabolites by the strain. We believe that using this approach could further increase the yield of diosgenin.

Subsequently, in investigating the effect of nitrogen sources on the ability of the SYt1 strain to catalyze the conversion of diosgenin from yam, this study demonstrated that a composite nitrogen source of yeast extract and peptone yielded the best conversion results. This finding is consistent with other studies related to Bacillus species. For instance, a mixture of yeast extract and soybean powder is the optimal nitrogen source for Bacillus subtilis, and a mixture of ammonium sulfate and peptone is the best nitrogen source for *Bacillus amyloliquefaciens* B128 [23,24,25]. Therefore, we believe that using a composite nitrogen source is more conducive to the metabolic activities of the endophytic *Bacillus licheniformis* SYt1 compared to a single nitrogen source.

Fermentation conditions significantly influence microbial metabolic activities. This study also found that a neutral environment is most conducive to the biotransformation activities of SYt1. Similar to our results, previous research has shown that the optimal initial fermentation pH for Bacillus strains such as SYSU G01002T and the strain HNA-14(T) is within the neutral range [26,27]. Acidic or alkaline environments negatively impact microbial growth. Regarding substrate concentration, our study found that an appropriate concentration of diosgenin not only reduces substrate wastage but also ensures sufficient substrate availability for decomposition. Inorganic salts are crucial for constructing cell bodies, enzymes, and other structures. Inorganic salts also regulate a medium pH, osmotic pressure, and redox potential. Studies have shown that the activity of Bacillus strains is influenced by inorganic salt concentration, with a concentration of 2.5 g/L enhancing the cell activity and growth rate of Bacillus subtilis [28]. In our study, we found that adding 1.81 g/L of K_2_HPO_4_ effectively improved the conversion efficiency of SYt1. The inoculum age directly affects the microbial fermentation cycle. If the inoculum volume is too large, nutrient and oxygen availability will be insufficient as bacteria proliferate, leading to metabolic abnormalities and affecting product synthesis [29]. Conversely, too little inoculum wastes resources and prolongs the lag phase, extending the fermentation period and significantly increasing costs [30]. Our study found that a seed age of 24 h is optimal for subsequent fermentation processes.

The Plackett–Burman experimental design method can quickly and effectively identify significant factors among multiple variables [31]. However, it can only detect factors with significant effects and cannot describe interactions between factors [32]. In contrast, response surface methodology (RSM) can identify the relationships between factors [33,34]. In this study, using the Plackett–Burman design, we found that peptone, yeast extract powder, and inorganic salt concentration were the main factors influencing the production of diosgenin by SYt1. Subsequent optimization using RSM can help in understanding the interactions between these factors and determining the optimal conditions for maximum production. The medium conditions were optimized by response surface design by Chen et al. to increase the spore production of B. subtilis WHK Bacillus from 2.7 ± 0.75 × 10^9^ to 1.52 ± 0.06 × 10^10^ CFU/mL [34]. Sun et al. used RSM to optimize the fermentation conditions of Pichia pastoris GS115 to increase xylanase production, resulting in a 2.1-fold increase in yield [35]. In this study, RSM was employed to determine the optimal concentrations of the key nutritional factors affecting the transformation efficiency of *Bacillus licheniformis* SYt1, specifically yeast extract, peptone, and dipotassium hydrogen phosphate. After optimizing the culture medium, the transformation efficiency increased by 80%. These results demonstrate that RSM is an effective approach for medium optimization.

In this study, a new optimized medium was proposed, whereby the conversion efficiency of endophytic *Bacillus licheniformis SYt1* liquid fermentation was enhanced. A series of analytical methods, including TLC, HPLC, IR, and NMR, confirmed the presence of diosgenin in the fermentation broth. The optimization of carbon and nitrogen sources revealed that sucrose and peptone were the most effective in enhancing diosgenin yield, with the highest yield achieved at 86.93 mg/L when a combination of 25 g/L sucrose and 10 g/L dextrin was used. The use of the Plackett–Burman and Box–Behnken designs identified key factors influencing diosgenin production, and the optimized medium composition increased the diosgenin yield to 132.57 mg/L, representing an 80% improvement compared to pre-optimization conditions. These findings provide a strong foundation for scaling up the fermentation process for industrial applications. Moreover, it is thought that the conversion efficiency of the current strain of endophytic B. licheniformis SYt1 can be enhanced. For instance, it is possible to enhance the conversion rate by modifying the oxygen content. It has been demonstrated in some studies that an increase in the concentration of dissolved oxygen can result in an acceleration of the growth rate of Bacillus. Further increases in diosgenin yield may be achieved through the application of UV mutation breeding or the creation of genetically engineered bacteria. By analyzing the gene fragments that are primarily responsible for the transformation of endophyte SYt1 and the main transforming glycosidases, an attempt is being made to increase the yield of diosgenin by directly producing the glycosidases required for production through plasmid construction.

## Figures and Tables

**Table 1:** P-B experimental design.

Symbol	Factor	Actual Experimentation Value	
		Low (−1)	High (1)
A	Yeast extraction (g/L)	15	25
B	Peptone (g/L)	10	20
C	Virtual item1	-	-
D	Sucrose (g/L)	5	15
E	Dextrin (g/L)	10	20
F	Virtual item2	-	-
G	*Dioscorea* rhizome powder (g/L)	3	8
H	Fermentation period (day)	4	6
J	Virtual item3	-	-
K	Inorganic salt concentration (g/L)	1	3
L	Inoculum age (h)	18	30

**Table 2:** Factors and their levels examined in the BBD.

Variables	Symbol	Levels		
		−1	0	+1
Peptone (g/L)	A	25	37.5	50
Yeast extract (g/L)	B	10	15	20
Inorganic salt (g/L)	C	1	2	3

**Table 3:** P-B design results.

	Sum of		Mean	F	*p*-Value
**Source**	Squares	df	Square	Value	Prob > F
**Model**	298.33	8	37.29	9.87	0.043
**Yeast infusion powder (g/L** **)**	85.33	1	85.33	22.59	0.0177
**Peptone (g/L** **)**	108	1	108	28.59	0.0128
**Sucrose (g/L** **)**	8.33	1	8.33	2.21	0.2342
**Dextrin (g/L** **)**	1.33	1	1.33	0.35	0.5943
**Inducer (g/L** **)**	0.33	1	0.33	0.088	0.7858
**Time (d** **)**	40.33	1	40.33	10.68	0.0469
**Inorganic salt (g/L)**	33.33	1	33.33	8.82	0.059
**Inoculum age (h** **)**	21.33	1	21.33	5.65	0.0979
**Residual**	11.33	3	3.78		
**Cor Total**	309.67	11			

**Table 4:** BBD experimental variance results.

	Sum of		Mean	F	*p*-Value
**Source**	Squares	df	Square	Value	Prob > F
**Model**	3840.714706	9	426.7461	34.31617	<0.0001
**A**	136.125	1	136.125	10.9463	0.0130
**B**	105.125	1	105.125	8.453475	0.0227
**C**	84.5	1	84.5	6.794945	0.0351
**AB**	121	1	121	9.73004	0.0169
**AC**	56.25	1	56.25	4.523262	0.0710
**BC**	6.25	1	6.25	0.502585	0.5013
**A^2**	1125.568421	1	1125.568	90.51096	<0.0001
**B^2**	1496.094737	1	1496.095	120.3063	<0.0001
**C^2**	388.0421053	1	388.0421	31.20385	0.0008
**Residual**	87.05	7	12.43571		
**Lack of Fit**	56.25	3	18.75	2.435065	0.2049
**Pure Error**	30.8	4	7.7		
**Cor Total**	3927.764706	16			

## Data Availability

The original contributions presented in the study are included in the article, further inquiries can be directed to the corresponding author.
